# The layer of lesson preparation delegated to AI predicts classroom discourse quality: a four-wave video study of 90 junior secondary mathematics teachers

**DOI:** 10.3389/fpsyg.2026.1962005

**Published:** 2026-10-06

**Authors:** Hongyu Chen, Na Wang, Chong Chen, Dirong Zhang, Xin Dang

**Affiliations:** 1 Northeast Normal University, Changchun, China; 2 Fuyang Normal University, Fuyang, China; 3 Guizhou Normal University, Guiyang, China; 4 Rikaze Vocational Technical College, Rikaze, China

**Keywords:** classroom discourse, cognitive offloading, confidence-performance dissociation, generative artificial intelligence, lesson preparation, mathematics education, multilevel mediation, teacher noticing

## Abstract

Research on teachers and generative artificial intelligence has largely measured how much they use these tools, leaving open which cognitive operations they hand over. We asked whether the layer of processing delegated during lesson preparation predicts what happens in the classroom. Ninety junior secondary mathematics teachers in 18 public schools in eastern China were followed across four waves of video-recorded lessons over one school year, yielding 897 coded lessons, 4,640 unplanned student contributions, 1,518 preparation sessions, and 556 stimulated-recall interviews. For each lesson, the final plan was compared against the interaction logs segment by segment, producing two ratios that index offloading by provenance: generative-layer offloading, the share of questioning and variation segments originating in model output, and formulation-layer offloading, the share of context and wording segments. Three-level models decomposing within- and between-teacher variation showed that the two layers behaved differently. Within teachers, generative-layer offloading was associated with less higher-order questioning, less substantive uptake of student talk, weaker maintenance of high cognitive demand, and lower recognition and substantive use of unplanned student contributions, with standardized coefficients between 0.08 and 0.18. Formulation-layer offloading was unrelated to four of those indicators, positively associated with the length and variety of designed variation sequences, and positively associated with recognition of unplanned contributions, the single indicator on which the two layers cross in opposite directions. Total preparation time with AI predicted discourse outcomes on its own and lost that value for the lesson-level indicators once layer composition was entered. An embedded micro-randomized comparison, in which teachers drafted their own questioning and variation sequence before consulting the model, lowered generative-layer offloading and raised substantive uptake. Professional attention to student thinking, coded from stimulated-recall interviews conducted after the lesson by a separate team, followed the same within-teacher pattern and carried the association with the substantive use of unplanned contributions. However, the retrospective measurement leaves its direction open. Teachers’ post-preparation confidence rose with generative-layer offloading by 0.23 standard deviations per within-teacher standard deviation. It carried no positive information about the lesson that followed, a confidence-performance dissociation. What predicted classroom discourse was which layer was delegated, not how much.

## 1 Introduction

Lesson preparation is where generative artificial intelligence has entered teachers’ work most visibly. In a mid-year implementation report covering 2,891 active teachers, preparation functions accounted for between 60 and 99% of platform use in every participating district (Esbenshade et al., 2026). Surveys of teachers who have adopted these tools report the same concentration in Chinese secondary schools and in Israeli ones (Tang and Zhong, 2026; Hazzan-Bishara et al., 2025). The majority of research that followed this development has asked whether teachers will adopt these systems and how heavily they will use them.

Those questions have been answered at length. A meta-analysis of technology acceptance research in artificial intelligence in education, covering 27 studies and more than 12,000 participants, found the pooled evidence resting on five constructs: perceived ease of use, perceived usefulness, attitude, behavioral intention, and self-reported use. Only five of the 27 studies measured use, and the meta-analytic model contains no variable describing teaching (Xue et al., 2026). Systematic reviews of generative AI in K-12 settings report a similar boundary. Four independent reviews with partly overlapping search windows synthesized 45, 130, 30, and 39 studies, and across all of them, none used classroom discourse quality as an outcome. In one, the terms classroom discourse and lesson planning appear only in reference titles; in another, a single included paper examined how teachers actually enacted the technology (Zhang et al., 2026; Marzano, 2026; Alfarwan, 2025; Costache et al., 2026). Teachers use these systems when they design lessons, and we know little about what the lessons look like afterward.

A second body of literature asks what delegation costs. Its conclusions run in the opposite direction: self-reported AI use covaries with self-reported offloading, and offloading covaries negatively with critical thinking (Gerlich, 2025a, corrected in Gerlich, 2025b). The independent variable there is a quantity. Offloading is measured as frequency or degree of reliance, and the taxonomy in the field’s foundational review is organized by where cognition is offloaded to, whether the body, the world, or other people, leaving open whether different forms of offloading are cognitively equivalent (Risko and Gilbert, 2016). Later theoretical work sharpened the mechanism without changing the grain: offloading behaves like a value decision, lowering the worth of remembering more than the capacity to remember (Gilbert, 2024), and it redirects representation from content toward index (Sparrow et al., 2011).

Several results suggest that grain is what matters. In two randomized online experiments, an assistant that supplied finished answers and one that supplied methods elicited statistically indistinguishable amounts of help-seeking, yet transfer performance differed between the two, and supplying answers first predicted worse transfer (Spatola, 2024). Labeled provenance, by contrast, appears inert: in a preregistered experiment with 295 participants, describing guidance as machine-generated rather than human-generated changed nothing on agreement, accuracy, sensitivity, or response bias, while whether the guidance was correct made a large difference to what participants concluded (Pearson et al., 2026). Overreliance of this kind is a behavior, and it is diagnosable only where the person retained the competence to judge otherwise (Ibrahim et al., 2025). Neither the amount of assistance taken nor its labeled source looks like the operative variable.

Applying this to preparation requires a principled partition of the preparatory work. The multimedia learning tradition supplies one. Generative processing consists of organizing incoming material into a coherent structure and integrating it with prior knowledge, and selection is explicitly excluded from it (Fiorella and Mayer, 2016; Mayer, 2024). Extraneous processing is imposed by the presentation of material, and much of instructional design exists to minimize it (Sweller et al., 2019; Sweller, 2023). Transposed to preparation, these give two operations of different theoretical standing. Composing the wording of an explanation and assembling contextual materials are handling costs, and delegating them should free capacity. Designing the questioning sequence and ordering the variations through which a concept will be encountered is organizing and integrating. We call these the formulation layer and the generative layer. The partition is proposed as a functional taxonomy rather than as a claim that either category is cognitively homogeneous: the wording of an explanation can carry considerable pedagogical content knowledge, and a familiar question sequence may cost an experienced teacher very little to produce. The distinction depends on whether the two layers behave differently, which is an empirical question.

Whether preparation admits such a partition is itself contested. One recent review groups planning with grading among the low-level cognitive tasks that machines may reasonably assume (García Espinoza et al., 2026). Against that, the CODE-PLAN model decomposes preparation into six empirically distinguishable cognitive demands validated on several hundred lesson plans (König et al., 2021); lesson-study research finds that most of collaborative preparation time goes to deliberating the flow of the research lesson, with tasks newly built or substantially adapted (Fujii, 2016); and the five-practices account describes the central benefit of preparation as expanding the time available for an instructional decision from seconds to minutes or hours (Stein et al., 2008). Conceptual work on the limits of delegation converges from another side, arguing that teaching tasks carry information for one another and resist modularization (Han, 2026), and that deskilling is better understood as the erosion of particular cognitive practices than as wholesale loss of expertise (Rind, 2026).

Classroom discourse is the outcome that neither literature has reached. Preparation research established a path from planning competence to student-rated instructional quality but found that none of its six dimensions predicted cognitive activation, the construct closest to questioning demand (König et al., 2021). Discourse research moved the other way: a large dialogic teaching trial whose explicit object was the transformation of classroom talk measured attainment, and its developer later recorded that the absence of an agreed measure of spoken language left the intervention untraceable to its own target (Jay et al., 2017; Alexander, 2018). Instruments that can close the gap exist. Intellectual challenge and uptake of student responses are separately scored components of the PLATO protocol (Klette et al., 2025); international video work anchors a questioning scale between recalling and reporting answers at one pole and analyzing, synthesizing, arguing, and conjecturing at the other (OECD, 2020). Uptake has a compact operational definition: student ideas are heard, taken up, and jointly considered (Mercer and Howe, 2012), elaborated into countable categories elsewhere (Resnick et al., 2015).

If generative-layer offloading reaches classroom discourse, professional attention to student thinking is the most plausible carrier. Across four contrast groups, attending, interpreting, and deciding how to respond separate cleanly, and deciding is the one component that teaching experience alone does not confer (Jacobs et al., 2010). Competence models place these situation-specific skills between stable dispositions and observable performance (Blömeke et al., 2015), and video-club research provides a scheme for classifying the object of a teacher’s attention and detecting its redistribution (Sherin and van Es, 2009; van Es and Sherin, 2021). Recent conceptual analysis adds a design implication: expertise differences in attention are clearest in non-aligned situations, where events depart from the anticipated course (Seidel et al., 2026). The moment a student says something the plan did not anticipate is thus the measurement point with the most discriminating power, and the same moment is where the five-practices account expects prior preparation to pay off (Stein et al., 2008).

One further element is needed to explain why teachers might not detect such a cost. Manipulating only the fluency of a lecture, with content held constant, moves judgments of learning by about a standard deviation while leaving actual recall unchanged (Carpenter et al., 2013), and the misattribution survives repeated study and test cycles with feedback (Yue et al., 2013). Reviews in this tradition state that fleeting gains during acquisition are likely to mislead instructors as well as students (Soderstrom and Bjork, 2015; Bjork et al., 2013), and that material one could have generated but was instead given represents a forgone learning opportunity (Bjork and Bjork, 2011). The participants in this work are overwhelmingly learners; teachers appear mainly as recipients of advice. Work in human-computer interaction predicts the same asymmetry for generative systems, holding that greater offloading raises metacognitive demand and that processing fluency serves as a misleading cue (Tankelevitch et al., 2024). Foresight analysis of teaching anticipates that seamless systems will lead teachers to mistake the system’s suggestions for their own decisions (Biström and Mollwing, 2026).

We joined these strands in one design. Ninety junior secondary mathematics teachers were tracked across four waves of video-recorded lessons over a school year. Offloading was measured separately at the two layers by segment-by-segment comparison of final plans against interaction logs. Classroom discourse was coded from transcripts; each unplanned student contribution and the teacher’s response were recorded. Professional attention was measured through independently conducted stimulated-recall interviews, and a micro-randomized comparison was embedded in the second half of the year. We expected the two layers to behave differently, with generative-layer offloading associated with weaker discourse indicators and formulation-layer offloading either neutral or, where freed capacity matters, positive. We expected total volume of AI use to lose predictive value once layer composition was modeled, the association with real-time responsiveness to run through attention to student thinking, and teachers’ subjective preparedness to track generative-layer offloading more closely than any observable feature of the lesson.

## 2 Materials and methods

### 2.1 Design and setting

The study used a dose–response tracking design with an embedded within-teacher micro-randomized comparison in the two later waves. A binary contrast between users and non-users was not feasible, because AI-assisted preparation tools are already widespread in the study region and no control condition could be insulated from them. In a two-week preparation diary kept before the study began, in early September 2025, teachers recorded generative AI assistance for 37.4% of their new-teaching lessons. The independent variable is therefore the continuous, lesson-varying composition of what is delegated, and the primary estimand is the within-teacher, between-lesson association obtained by decomposing each offloading variable into its teacher mean and its teacher-mean-centered deviation.

Four observation waves were scheduled across the 2025 to 2026 school year: a baseline wave from 15 September to 17 October 2025, a post-workshop wave from 24 November to 22 December 2025, a mid-year wave from 2 March to 10 April 2026, and an end-of-year wave from 18 May to 15 June 2026. Ninth-grade new instruction concludes in late April before examination revision begins, so the end-of-year window for ninth-grade teachers moved to 8 to 30 April 2026, and their topics were restricted accordingly. Windows for other grades did not overlap. Between the first two waves, teachers attended a four-week tiered workshop on human-AI collaborative preparation and prompting strategies. The workshop contained no classroom discourse training, and participation hours were self-selected (8, 16, 24, or 32 h; 20, 27, 25, and 18 teachers). Because participation was self-selected and hours showed no stable association with wave-to-wave change in any discourse indicator, workshop dosage is treated as a descriptor of selective engagement and is not analyzed as a dose.

Scheduling was constrained by curriculum sequence. Lesson topics were bound to the actual teaching window of the corresponding chapter in the national textbook series; teaching dates could fall only within the intersection of that chapter window and the observation window; no teacher taught the same topic twice across the study; and chapter order was never reversed across waves. Two lesson-period systems, of 40 and 45 min, coexist among local schools and are recorded per school.

### 2.2 Participants

Ninety mathematics teachers were recruited from 18 public junior secondary schools across five districts and counties of one prefecture-level city in eastern China, covering urban, peri-urban, and county school types (8, 5, and 5 schools). School enrollment ranged from 677 to 2,465 students, and district digital education ratings ranged from 2 to 4 on a five-point scale. Teachers averaged 13.52 years of experience (SD = 7.67), and 58.9% were women; 82.2% held a bachelor’s degree, 15.6% a master’s, and 2.2% an associate degree. Professional ranks were distributed across unranked or grade three (1), grade two (31), grade one (40), and senior (18). Grade assignments were balanced across grades seven, eight, and nine (33, 29, and 28 teachers). Each teacher taught one or two mathematics classes, for a total of 165 classes. Averaged over lessons, registered class size was 45.41 students (SD = 4.00); averaged over classes, it was 45.09 (SD = 4.11). Table 1 reports sample characteristics.

**Table 1:** Sample characteristics.

Characteristic	Value
Schools	18 (urban 8, peri-urban 5, county 5)
Enrollment	677 to 2,465 students
District digital education rating (1–5)	2 (*n* = 2), 3 (*n* = 8), 4 (*n* = 8)
Lesson period system	40 min (*n* = 10) or 45 min (*n* = 8)
Teachers	90
Years of experience	M = 13.52, SD = 7.67, range 2 to 35
Women	58.9%
Highest degree	Bachelor 82.2%, master 15.6%, associate 2.2%
Professional rank	Senior 18, grade one 40, grade two 31, unranked or grade three 1
Grade taught	Grade 7 (33), grade 8 (29), grade 9 (28)
Self-selected workshop hours	8 (20), 16 (27), 24 (25), 32 (18)
Pre-study share of lessons prepared with AI	37.4% (two-week diary, early September 2025)
Withdrew before wave 4	9
Classes taught	165 (1 or 2 per teacher)
Registered class size	M = 45.41, SD = 4.00
Lessons scheduled	970
Valid for discourse coding	897 (92.5%)
By wave	239, 231, 216, 211
Scheduled teacher-by-wave cells with 3/2 lessons	290/50
Students present	M = 43.72, SD = 4.20
Actual lesson duration (min)	M = 40.91, SD = 2.94
Unplanned student contributions	4,640 events in 861 lessons
Human-AI preparation sessions	1,518 sessions in 905 lessons
Stimulated-recall interviews	556 (62.0% of valid lessons)
Double-coded lessons	179 (20.0%)

Counts in parentheses are numbers of teachers unless otherwise stated. Registered class size is averaged over lessons; averaged over the 165 classes, it is 45.09 (SD = 4.11). Session logs cover 905 lessons because preparation records exist for some lessons whose video coding later failed.

A total of 970 lessons were scheduled, of which 897 (92.5%) yielded valid discourse coding. Exclusions included rescheduling or teacher absence (43), audio recorder failure (11), camera obstruction that prevented complete coding (8), lessons that inspection showed were review rather than new instruction (8), and unusable ambient noise (3). Valid lessons per wave were 239, 231, 216, and 211. Ninety teachers across four waves define 360 teacher-by-wave cells, of which 340 were scheduled; the 20 remaining cells belong to the nine teachers who withdrew. Of the 340 scheduled cells, 290 contained three lessons, and 50 contained two, reflecting the number of new-teaching periods available within the relevant chapter window; the counts above therefore describe the scheduled sample, not the coded one. Nine teachers withdrew before completing all four waves due to maternity leave, long-term sick leave, inter-school transfer, or voluntary withdrawal.

### 2.3 Ethics approval and consent

The study was approved by the ethics committee of Rikaze Vocational Technical College. All participating teachers gave written informed consent. The lessons were recorded in eastern China, where the authors had existing working relationships with the participating schools, and all data were collected by the authors themselves. Ethical review was carried out at the home institution of one of the authors, outside the study region. Class-level guardian consent for student recording was obtained at 97.4%, and the contributions of students whose guardians declined were neither transcribed nor coded. Those students remain in the registered class size but are excluded from the count of public contributions, which slightly reduces the denominator of the uptake measure in a way that is unrelated to teacher preparation. Schools, teachers, students, and AI platforms are represented throughout by codes or category labels, and place names are generalized.

### 2.4 Measures

#### 2.4.1 Offloading layers

Every preparation session with a generative AI system was logged, giving 1,518 sessions across 905 lessons. Logs recorded start time, duration, number of prompt-response turns, platform category, dominant request type, how output entered the final plan, and the semantic similarity between session output and the corresponding segment of the finalized plan. Output entered the plan by direct adoption in 51.2% of sessions, with retained structure and rewriting in 23.1%, key points only in 18.8%, and no adoption in 6.9%. Mean semantic similarity to the final plan was 0.64 (SD = 0.28), and mean turns per session was 4.65 (SD = 3.46).

Offloading was measured by segment-by-segment comparison of the finalized plan against these logs. Each plan was partitioned into segments, and segments were classified as belonging either to questioning logic and variation sequencing or to contextual material and linguistic formulation. Generative-layer offloading is the proportion of the former judged on comparison to originate directly in model output, with a mean denominator of 11.38 segments; formulation-layer offloading is the corresponding proportion of the latter, with a mean denominator of 11.28. The criterion for origin is which party performed the organizing operation. A segment counts as originating in model output when the ordering of questions or the sequence of variations derives from model output, whether or not the teacher rewrote the wording. Output whose structure was retained under rewriting therefore counts; output that was not adopted does not; and output from which the teacher took only key points was judged segment by segment, counting when the points themselves supplied the sequence and not counting where the teacher assembled it. The manner in which output entered the plan is a session-level label, and one session can contribute several segments, so it does not by itself determine the count. Semantic similarity was computed after the origin judgments were complete and was not available to the judges; it is a descriptive quantity, and no threshold was applied at any stage. Both are count ratios. The two ratios correlated only weakly (*r* = 0.21), suggesting separable practices instead of a single tendency to delegate. Judges were blind to the discourse coding, the coding of unplanned contributions, and every outcome for the lesson concerned, and the offloading judgments were completed before discourse coding began. The rule manual was finalized before the first judgment and was not revised thereafter. A second researcher independently re-judged a random 180 lessons (20.1% of the valid sample). Agreement was ICC(2,1) = 0.904 for the number of segments after partitioning, Cohen’s kappa = 0.86 for layer classification, and Cohen’s kappa = 0.79 for the origin judgment; disagreements were arbitrated by a third researcher, and the arbitrated values entered the analyses. The two judges’ generative-layer ratios correlated at *r* = 0.92, with a mean absolute difference of 0.041. Semantic similarity, computed independently and not seen by the judges, separated the origin categories with an area under the curve of 0.87 and rose monotonically across the manner of adoption, from 0.12 where output was not adopted to 0.30, 0.61, and 0.86. The ratios record where plan content originated rather than how much processing the teacher performed.

#### 2.4.2 Classroom discourse

All lessons were transcribed from audio and human-verified, then coded by six trained coders using a common manual. Teacher questions were assigned to one of four cognitive levels, with the upper two combined as higher-order questions; the indicator is the proportion of higher-order questions among all questions asked. Coding was informed by classroom analyses, at the same phase of schooling, of how teachers follow up on student contributions with questions that stimulate productive thinking (Chin, 2007). Substantive uptake follows the definition of student ideas being heard, taken up, and jointly considered (Mercer and Howe, 2012) and counts teacher moves that probe, reformulate, or extend a student contribution, excluding evaluative acceptance; the indicator divides these moves by the number of student public contributions. Maintenance of cognitive demand is the number of planned high-demand tasks that were not reduced to procedural exercise during enactment, divided by the number planned. Every lesson had at least one planned high-demand task, with denominators ranging from 1 to 6 (modal value 2), so no denominator is zero.

Variation structure was coded as chains, following the requirement that variation be discernible only against a background of relative invariance and the caution that too many simultaneously varying dimensions leave nothing to be discerned (Watson and Mason, 2006). A chain contains at least two variations, and isolated variations are not counted. Chains were classified into four operational types according to what is held invariant and what is varied: conceptual variation, in which the representation of an object changes while the object does not; procedural variation, in which the route to a solution changes; contextual variation, in which the setting or the referent changes; and conditional variation, in which a given or a constraint changes. We report the number of chains, the length of the longest chain, the number of types present, and, for the by-type analysis in Section 3.3, the number of individual variation items within each type. The fourfold partition is an operational segmentation of variation structure. It sits within the tradition of teaching through variation in Chinese mathematics classrooms (Gu et al., 2017; Pang et al., 2017) but does not claim correspondence to any of the categories established there. A normalized diversity index derivable from these counts is zero for the roughly four lessons in 10 that used a single type; it is reported descriptively, and the number of types serves as the ordinal indicator.

#### 2.4.3 Unplanned student contributions

Every student contribution that departed from the anticipated course of the lesson was recorded as a discrete event, giving 4,640 events across 861 lessons. Such events are a proper subset of student public contributions. Each event carries its onset time from the start of the lesson, a type classification into five categories, namely typical errors, challenges or probing questions, anticipatory questions, appeals to everyday experience, and divergent associations, the seat number of the contributing student, whether the teacher responded in any form, and a four-level response code: 0 for not recognized or ignored, 1 for procedural acceptance, 2 for surface restatement, and 3 for substantive use that advanced the lesson. This event stream operationalizes the non-aligned situation identified as the point of maximum discriminating power for expertise (Seidel et al., 2026) and as the payoff point for prior preparation (Stein et al., 2008). The same terminology is used throughout: recognition and substantive use of unplanned contributions.

#### 2.4.4 Professional attention

Professional attention was measured using stimulated recall, a retrospective method developed because pre-lesson goals alone do not account for classroom conduct (Calderhead, 1981; Gass and Mackey, 2017). Each valid lesson had approximately a 60% probability of being sampled for interview, independent of its sequence number, yielding 556 interviews covering 62.0% of valid lessons. Teachers reviewed segments of the recently taught lesson and commented freely. Transcripts were segmented into statement units (a mean of 44.8 per interview), and each unit was coded as addressing students’ thinking, the progression of instructional stages, the teacher’s performance, or content and materials. Interviews were conducted by four interviewers on a rotating basis, and no interviewer coded an interview that they had conducted. The indicator is the proportion of statements focused on student thinking (M = 0.38, SD = 0.18). It correlated with substantive uptake (*r* = 0.47), and the two were produced by different teams from different materials: a retrospective verbal report coded by someone other than the interviewer, against a count of real-time conversational moves coded from video. The interview follows the lesson it concerns.

#### 2.4.5 Subjective preparedness

On completing preparation and before teaching, teachers rated their confidence in handling student departures from the plan on three items, averaged on a seven-point scale (M = 4.33, SD = 0.94, available for 869 lessons). We report it as a subjective judgment; its relation to observed practice is examined in section 3.6.

#### 2.4.6 Covariates and additional records

Models include topic difficulty, a fixed property of each topic rated in advance by two subject specialists on a five-point scale; lesson type, ordinary or open demonstration lesson; and wave, entered linearly. Lesson, teacher, and school records also carry class size, attendance, lesson duration, weekday and period, grade, coder identity, teacher demographics and workload, school locale, enrollment, and district digital rating. These are described in the data dictionary identified in the data availability statement and are used in the missing-data model and in the sensitivity analyses reported below, not as routine adjustments in the primary models, which are kept deliberately sparse.

A 12-item situational judgment task on teaching scenarios was administered as a candidate moderator (0 to 3 points per item, M = 16.70, SD = 7.74; Cronbach’s alpha = 0.826; retest *r* = 0.677 in a random subsample of 30 teachers after 27 to 31 days). Assessment of adaptive expertise by one-shot scenario judgment has been argued on definitional grounds to miss the construct, which requires observing performance when learning opportunities and resources are supplied (Schwartz et al., 2005), and no instrument in this area reports reliability evidence for the construct itself (Hatano and Inagaki, 1986; Rousseau and Stouten, 2025). We therefore do not interpret the measure as adaptive expertise. Its interaction with within-teacher generative-layer offloading was not significant for higher-order questioning (*b* = 0.016, SE = 0.018, *p* = 0.375), and we draw no inference from that null.

### 2.5 Embedded micro-randomized comparison

In each of the two later waves, one lesson per teacher was randomly designated for a generative preservation protocol: the teacher drafted the questioning sequence and variation ordering unaided, and only then consulted the model, restricting its role to materials and formulation. This realizes in the field the contrast between supplying methods and supplying finished products (Spatola, 2024) and provides an exogenous shift in generative-layer offloading within teachers. Of 427 lessons in these waves, 154 were assigned to the protocol, and 130 implemented it, an implementation rate of 84.4%. No lesson implemented the protocol without being assigned to it, so the design is one-sided and the complier average causal effect is identified by the Wald ratio. All treatment estimates are reported as intention-to-treat. Realized minimum detectable effect sizes, computed as 2.8 times the estimated standard error divided by the outcome standard deviation, ranged from 0.18 to 0.29 standard deviations across outcomes.

### 2.6 Coder training and reliability

Six coders used a common manual, and 179 lessons (20% of the valid sample) were independently double-coded by a second coder, with pairings never repeating a coder within a lesson. Reliability for the offloading judgments is reported in section 2.4.1 and rests on a separate subsample of lessons. Double coding was motivated by the observation that rater effects account for a non-trivial share of variance in standardized classroom observation (Pianta and Hamre, 2009). At the level of individual conversational turns, chance-corrected agreement was Cohen’s kappa = 0.72 for question cognitive level, 0.75 for substantive response, and 0.69 weighted for variation chain identification. At the lesson level, single-rater agreement was ICC(2,1) = 0.887 for higher-order question counts, 0.901 for substantive response counts, and 0.839 for longest chain length. Because the analyses use the primary coder’s scores, ICC(2,1) is the operative coefficient; the corresponding two-rater averages, ICC(2,2) = 0.940, 0.948, and 0.912, are reported only for comparability with studies that analyze averaged scores. The gap between turn-level kappa and lesson-level ICC is an aggregation effect, since disagreements about individual turns partly cancel within a lesson.

Coders were distributed evenly across waves, with each coder handling between 22 and 50 lessons in each wave. To test for drift across the school year, primary models were refitted with coder fixed effects. Coefficients for within-teacher generative-layer offloading changed by less than 3% (higher-order questioning, from −0.103 to −0.100; substantive uptake, from −0.170 to −0.171), and the linear wave term retained its size and significance.

### 2.7 Analytic strategy

Lessons are nested within teachers within schools, and teachers who teach two classes generate a cross-classification of lessons by class. Omitting an intermediate level can inflate lower-level variance components and compress fixed-effect standard errors. In a longitudinal dataset of 8,367 students across 147 schools, dropping the class level inflated student-level variance by 17 to 23% (Gilbert et al., 2016). All models therefore specify random intercepts for school and for teacher. A class level cannot be estimated separately in this design. Each teacher taught one or two classes; 75 of the 90 taught exactly two, and each class contributes a mean of 5.4 lessons, so class is close to collinear with teacher. Fitting the four-level model confirms this. The partition of variance among school, teacher, and class is not identified: one component is driven to the zero boundary, the three shares change places across optimizers and between REML and ML estimation, and several specifications do not converge at all. The class level is therefore omitted because it cannot be separated from teacher, not because a stable estimate is small, and the fixed effects are unchanged to three decimal places under every specification we fitted. Unconditional models partitioned variance as 7.4% school and 25.3% teacher for higher-order questioning, 13.6 and 34.6% for substantive uptake, 3.2 and 11.4% for recognition of unplanned contributions, and 0.0 and 12.8% for longest chain length.

Each offloading variable was decomposed into its teacher mean and its teacher-mean-centered deviation, both entered simultaneously. This separates the within-teacher effect, which is free of stable teacher characteristics, from the between-teacher effect, which is not, and lets the difference between them index selection (Hox et al., 2018). All models include topic difficulty, lesson type, and wave. The three discourse proportions have lesson-varying denominators and are modeled as continuous outcomes, with binomial models retaining the denominators, count and ordinal models for the variation outcomes, and models entering wave categorically fitted as checks. Standardized coefficients are reported per standard deviation of the within-teacher predictor and per standard deviation of the outcome. Random slopes for within-teacher generative-layer offloading were tested for the three principal outcomes; slope variance was detectable for substantive uptake and for recognition of unplanned contributions, and random-intercept models are reported because the fixed effect moved by less than 4% in every case. In random-slope models, the fixed effect changes by 3.7% for substantive uptake and by 0.4% for recognition, while the standard error for substantive uptake rises by about a quarter without altering the inference. Slope variation across teachers is itself appreciable, at 0.139 and 0.169 against fixed effects of 0.170 and 0.251. Event-level analyses use an ordered logit for the four-level response code with the same decomposition, event type, and onset time as event-level covariates, and standard errors clustered by teacher. Mediation follows the multilevel framework for 1-1-1 designs (Preacher et al., 2010), with indirect effects tested by Monte Carlo confidence intervals from 200,000 draws. All models were estimated by maximum likelihood in Python 3.12 using statsmodels 0.14.6; all reported models converged. The 16 within-teacher tests are corrected using the Benjamini-Hochberg procedure. Estimates from the embedded trial and from the mediation models are reported without adjustment and are interpreted as one confirmatory test and one mechanism probe, respectively.

### 2.8 Missing data

Missingness has two identifiable channels. Teachers with heavier weekly loads were more likely to reschedule or be absent, and schools with lower district digital ratings were more likely to experience collection or coding failure; attrition is related to both. Among teachers who withdrew, mean weekly load was 12.11 periods against 10.83 for completers, and mean district digital rating was 2.78 against 3.46. Across all teachers, the number of valid lessons correlated −0.20 with weekly load and 0.46 with district digital rating. Observed data can rule out missingness completely at random but cannot establish missingness at random, which is an assumption rather than a finding; we assume missingness at random conditional on weekly load, district digital rating, and the other recorded covariates, and estimate using full-information maximum likelihood with those covariates included. Missing values are coded distinctly for not collected, not applicable, and coding unavailable, and no missing code enters any computation.

## 3 Results

### 3.1 Descriptive statistics

Across 897 valid lessons, generative-layer offloading averaged 0.345 (SD = 0.254) and formulation-layer offloading 0.548 (SD = 0.261). Preparation time with AI averaged 28.36 min per lesson (SD = 26.18, range 0 to 144). Teachers asked 34.43 questions per lesson (SD = 11.02), of which 23.5% were higher-order, and students made 28.56 public contributions (SD = 11.06), of which 29.1% drew a substantive teacher response. Of the 4,640 unplanned contributions, 61.3% were recognized in some form, and 10.3% were used substantively; the corresponding lesson-level means, which weight each lesson equally instead of each event, are 0.597 and 0.087. Table 2 reports descriptive statistics by wave. Refitting the primary models on the second judge’s offloading ratios moved the within-teacher coefficient for higher-order questioning from −0.103 to −0.099 and that for substantive uptake from −0.170 to −0.166. Under a narrower origin rule counting only verbatim direct adoption, generative-layer offloading averaged 0.223 (SD = 0.196) and correlated with the reported ratio at *r* = 0.90, giving within-teacher standardized coefficients of −0.15 and −0.20 against −0.14 and −0.18 under the reported rule.

**Table 2:** Descriptive statistics for valid lessons, overall and by observation wave.

Variable	Overall M (SD)	Wave 1	Wave 2	Wave 3	Wave 4
Generative-layer offloading	0.345 (0.254)	0.327	0.356	0.348	0.351
Formulation-layer offloading	0.548 (0.261)	0.470	0.545	0.585	0.601
AI preparation time (min)	28.36 (26.18)	23.62	28.77	30.58	31.02
Post-preparation confidence (1–7)	4.33 (0.94)	4.34	4.24	4.42	4.36
Total questions	34.43 (11.02)	34.45	34.86	34.61	33.74
Higher-order questioning (proportion)	0.235 (0.126)	0.207	0.217	0.246	0.273
Student public contributions	28.56 (11.06)	29.57	28.69	27.38	28.46
Substantive uptake (proportion)	0.291 (0.162)	0.264	0.277	0.306	0.321
Cognitive demand maintained	0.511 (0.370)	0.451	0.493	0.587	0.520
Unplanned contributions per lesson	5.39 (3.68)	5.67	5.42	5.15	5.28
Recognized (lesson-level proportion)	0.597 (0.289)	0.602	0.623	0.590	0.567
Used substantively (lesson-level)	0.087 (0.153)	0.094	0.094	0.079	0.082
Longest variation chain	3.64 (1.50)	3.57	3.64	3.72	3.66
Variation types used	1.52 (0.73)	1.55	1.49	1.56	1.48
Variation chains	2.15 (1.35)	2.16	2.21	2.17	2.07

*n* = 897 lessons, except for post-preparation confidence (*n* = 869) and the three indicators derived from unplanned contributions (*n* = 861 lessons). The two rows labeled lesson-level average lesson-level proportions and so weight each lesson equally; the corresponding event-level rates, pooling all 4,640 events, are 0.613 and 0.103. Wave columns report means.

Two features of Table 2 bear on interpretation. Formulation-layer offloading rose steadily across the year, from 0.470 to 0.601, while generative-layer offloading stayed close to flat, from 0.327 to 0.351, so the two indicators do not track a common escalation of AI use. Discourse indicators also improved across waves: higher-order questioning from 0.207 to 0.273 and substantive uptake from 0.264 to 0.321. The workshop between waves 1 and 2 included no discourse training, and the improvement continued after it, so the trend is unlikely to be a workshop effect. Reactivity to repeated observation, growing familiarity with the recording procedure, and coder drift are all plausible contributors. We tested the last of these directly, refitting the primary models with coder fixed effects; the wave term was unaffected, which weakens the drift account without excluding the other two. All primary estimates are within-teacher and include a linear wave term, so the trend is absorbed rather than confounded with the offloading estimates.

### 3.2 Total volume of AI use

Entered alone, within-teacher variation in preparation time with AI predicted lower higher-order questioning (*b* = −0.0038 per additional 10 min, *p* = 0.017) and lower substantive uptake (*b* = −0.0061 per 10 min, *p* = 0.001). Both associations disappeared when layer composition was added: the coefficient for preparation time fell to −0.0013 (*p* = 0.516) for higher-order questioning and to −0.0016 (*p* = 0.462) for substantive uptake, while generative-layer offloading remained strongly negative in both models (*b* = −0.094 and −0.160, both *p* < 0.001). The same comparison extended to the remaining lesson-level outcomes yields the same result. Once both layers were entered, preparation time was unrelated to maintenance of cognitive demand and to the three variation indicators, and the layer coefficients moved by less than 6%. At the event level, the picture is not uniform. Preparation time did not predict recognition of unplanned contributions, but its between-teacher component predicted their substantive use (*b* = 0.017, *p* = 0.024), and the within-teacher generative-layer coefficient weakened from −1.075 to −0.781 when it was entered. For the lesson-level indicators, then, time spent with the tool predicts discourse only insofar as it correlates with what is being delegated. For the two event-level indicators, the claim does not hold in that form, and the component that survives is a between-teacher one, confounded with stable differences among teachers.

### 3.3 The two layers behave differently

Table 3 reports three-level models with within- and between-teacher decomposition, and Figure 1 displays the standardized within-teacher coefficients. Within teachers, generative-layer offloading was associated with lower higher-order questioning (*b* = −0.103, SE = 0.020, *p* < 0.001; *β* = −0.14), lower su*b*stantive uptake (*b* = −0.170, SE = 0.022, *p* < 0.001; *β* = −0.18), weaker maintenance of cognitive demand (*b* = −0.362, SE = 0.066, *p* < 0.001; *β* = −0.17), lower recognition of unplanned contributions (*b* = −0.251, SE = 0.054, *p* < 0.001; *β* = −0.15), and lower substantive use of those contributions (*b* = −0.066, SE = 0.030, *p* = 0.025; *β* = −0.08). Its coefficients for variation structure were not distinguishable from zero. These are small to moderate effects: a one standard deviation increase in a teacher’s generative-layer offloading relative to their own average corresponds to roughly a sixth of a standard deviation change in the discourse indicators.

**Table 3:** Three-level models predicting classroom discourse from offloading at two layers, with within- and between-teacher decomposition.

Outcome	Within generative *b* (SE)	*β*	Within formulation *b* (SE)	*β*	Between generative *b* (SE)	Between formulation *b* (SE)
Higher-order questioning	−0.103 (0.020)***	−0.14	0.012 (0.017)	0.02	−0.270 (0.032)***	0.010 (0.033)
Substantive uptake	−0.170 (0.022)***	−0.18	0.022 (0.019)	0.03	−0.416 (0.056)***	0.033 (0.056)
Cognitive demand maintained	−0.362 (0.066)***	−0.17	0.097 (0.058)	0.05	−0.632 (0.083)***	−0.074 (0.089)
Recognition of unplanned contributions	−0.251 (0.054)***	−0.15	0.139 (0.048)**	0.10	−0.483 (0.067)***	0.181 (0.070)*
Substantive use of unplanned contributions	−0.066 (0.030)*	−0.08	0.002 (0.026)	0.00	−0.175 (0.039)***	0.042 (0.040)
Longest variation chain	−0.405 (0.273)	−0.05	1.342 (0.241)***	0.18	−0.914 (0.382)*	1.757 (0.419)***
Variation types used	0.145 (0.140)	0.03	0.494 (0.124)***	0.14	−0.169 (0.164)	0.543 (0.178)**
Variation chains	0.343 (0.250)	0.04	0.672 (0.221)**	0.10	−0.184 (0.467)	0.460 (0.473)

*n* = 897 lessons from 90 teachers in 18 schools, except for the two outcomes derived from unplanned contributions (*n* = 861). Random intercepts for school and teacher. All models control for topic difficulty, lesson type, and wave. *β* is standardized per standard deviation of the within-teacher predictor (0.174 for generative, 0.201 for formulation) and per standard deviation of the outcome. *p < 0.05, **p < 0.01, ***p < 0.001. Under Benjamini-Hochberg correction across the 16 within-teacher tests, all nine significant coefficients retain *q* < 0.05, the weakest at *q* = 0.045.

**Figure 1:**
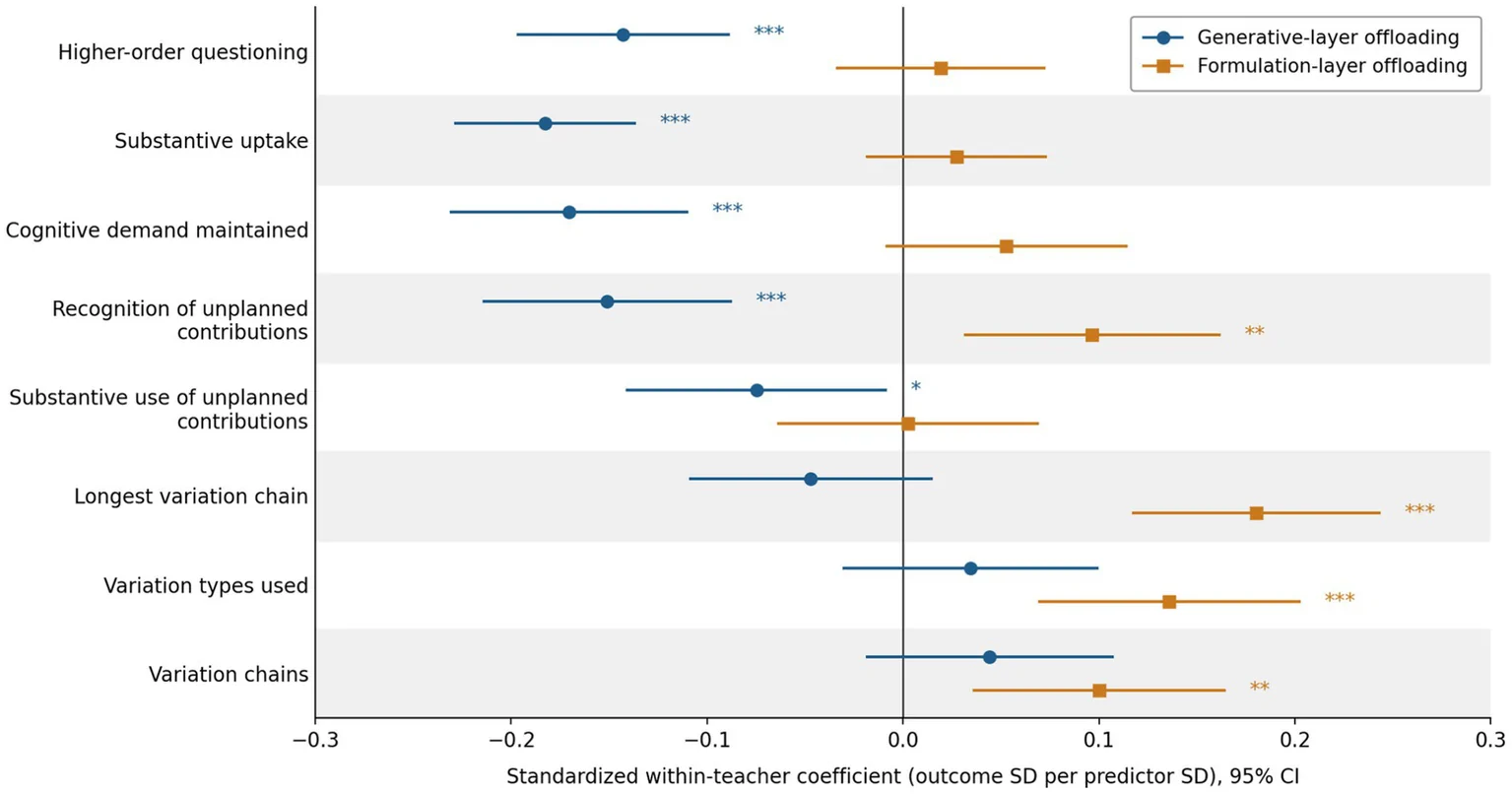
Standardized within-teacher associations between offloading at two layers and eight classroom discourse indicators. Points are regression coefficients from three-level models with random intercepts for school and teacher, standardized per standard deviation of the within-teacher predictor and per standard deviation of the outcome; horizontal bars are 95% confidence intervals. Circles denote generative-layer offloading and squares denote formulation-layer offloading. Asterisks mark significance of the unstandardized coefficient (*p < 0.05, **p < 0.01, ***p < 0.001). *n* = 897 lessons, except for the two indicators derived from unplanned contributions (*n* = 861).

Formulation-layer offloading produced a different pattern. It was associated with longer variation chains (*b* = 1.342, SE = 0.241, *p* < 0.001; *β* = 0.18), more variation types (*b* = 0.494, SE = 0.124, *p* < 0.001; *β* = 0.14), more chains overall (*b* = 0.672, SE = 0.221, *p* = 0.002; *β* = 0.10), and higher recognition of unplanned contributions (*b* = 0.139, SE = 0.048, *p* = 0.004; *β* = 0.10). For higher-order questioning, substantive uptake, and substantive use of unplanned contributions, the coefficients were small and not distinguishable from zero. The generative layer shows negative associations across five indicators and none for variation structure, while the formulation layer carries positive associations across the three variation indicators and for recognition and none elsewhere. The only indicator on which both layers reach significance, recognition of unplanned contributions, shows them in opposite directions.

For the five discourse indicators on which the generative layer carries negative within-teacher coefficients, the between-teacher coefficients are larger by factors between 1.75 and 2.65. For two of the variation outcomes, the two estimates differ in sign rather than in magnitude. Teachers whose preparation habitually leans on model-generated questioning differ from other teachers in ways beyond that habit, and the gap between the two estimates quantifies how much of a naive cross-sectional association would be attributable to who chooses to delegate. Our substantive claims rest on the within-teacher estimates. The pattern does not depend on the linear specification. Binomial models that retain the denominators give average marginal effects of −0.11, −0.18, and −0.36 for the three proportions, against linear coefficients of −0.10, −0.17, and −0.36; Poisson and ordinal models reproduce the formulation-layer associations with variation structure, all at *p* < 0.01; and entering wave categorically changes the offloading coefficients by less than 1%. Maintenance of cognitive demand is the one outcome whose wave trajectory is not monotonic, peaking at wave 3 (Table 2), and it is the outcome for which the categorical specification fits best. Decomposing the variation outcomes by type shows where the formulation-layer association sits. Counting individual variation items, the within-teacher coefficient for formulation-layer offloading is 1.72 for procedural variation (SE = 0.46, *p* < 0.001) and 1.27 for conditional variation (SE = 0.48, *p* = 0.008), against 0.81 for conceptual variation (SE = 0.49, *p* = 0.098) and 0.64 for contextual variation (SE = 0.51, *p* = 0.213). Counting chains rather than items preserves the ordering, with contextual variation the weakest of the four (*b* = 0.06, SE = 0.15, *p* = 0.699). Of the 16 within-teacher tests in Table 3, nine are significant: five for the generative layer and four for the formulation layer, and all nine survive the Benjamini-Hochberg correction; four of the five generative-layer results carry adjusted q below 0.001 and the fifth q = 0.045, while q for the formulation-layer results runs from below 0.001 to 0.007.

### 3.4 Responses to unplanned student contributions

Ordered logit models across all 4,640 events, with standard errors clustered by teacher, reproduced the pattern at the level of the individual student utterance (Table 4). Within teachers, generative-layer offloading lowered the odds of a higher response level (*b* = −1.391, SE = 0.153, *p* < 0.001), which corresponds to an odds ratio of 0.79 per within-teacher standard deviation of the ratio and 0.87 per 0.1 increment. Formulation-layer offloading trended in the opposite direction without reaching conventional significance (*b* = 0.274, SE = 0.157, *p* = 0.081), and its between-teacher component was positive (*b* = 0.641, *p* = 0.025).

**Table 4:** Ordered logit for teacher response level to unplanned student contributions.

Predictor	*b* (SE)	Odds ratio	*z*	*p*
Generative-layer offloading (within)	−1.391 (0.153)	0.249	−9.10	<0.001
Formulation-layer offloading (within)	0.274 (0.157)	1.316	1.74	0.081
Generative-layer offloading (between)	−2.342 (0.190)	0.096	−12.32	<0.001
Formulation-layer offloading (between)	0.641 (0.287)	1.899	2.24	0.025
Topic difficulty	−0.028 (0.047)	0.973	−0.59	0.557
Open demonstration lesson	0.003 (0.090)	1.003	0.03	0.977
Wave	−0.018 (0.025)	0.982	−0.74	0.461
Onset time within lesson	0.017 (0.035)	1.017	0.47	0.641
Type: challenge or probing	0.255 (0.092)	1.291	2.77	0.006
Type: everyday experience	−0.607 (0.078)	0.545	−7.75	<0.001
Type: anticipatory question	−0.723 (0.130)	0.485	−5.58	<0.001
Type: divergent association	−0.792 (0.090)	0.453	−8.78	<0.001

*n* = 4,640 events in 861 lessons taught by 90 teachers. Response level is coded 0 (not recognized or ignored), 1 (procedural acceptance), 2 (surface restatement), 3 (substantive use). Odds ratios are per unit of the predictor, that is, across the full 0 to 1 range of each ratio; the odds ratio per within-teacher standard deviation of generative-layer offloading is 0.79. Standard errors clustered by teacher. Reference category for event type is typical error. Threshold parameters omitted.

Event type mattered substantially and independently of preparation. Relative to typical errors, challenges and probing questions drew higher responses (*b* = 0.255, *p* = 0.006), whereas divergent associations (*b* = −0.792), anticipatory questions (*b* = −0.723), and appeals to everyday experience (*b* = −0.607) drew markedly lower ones (all *p* < 0.001). Descriptively, 71.6% of challenges were recognized, and 17.5% were used substantively, compared with 50.7 and 5.2% for divergent associations (Figure 2). Onset time within the lesson, topic difficulty, lesson type, and wave were unrelated to response level.

**Figure 2:**
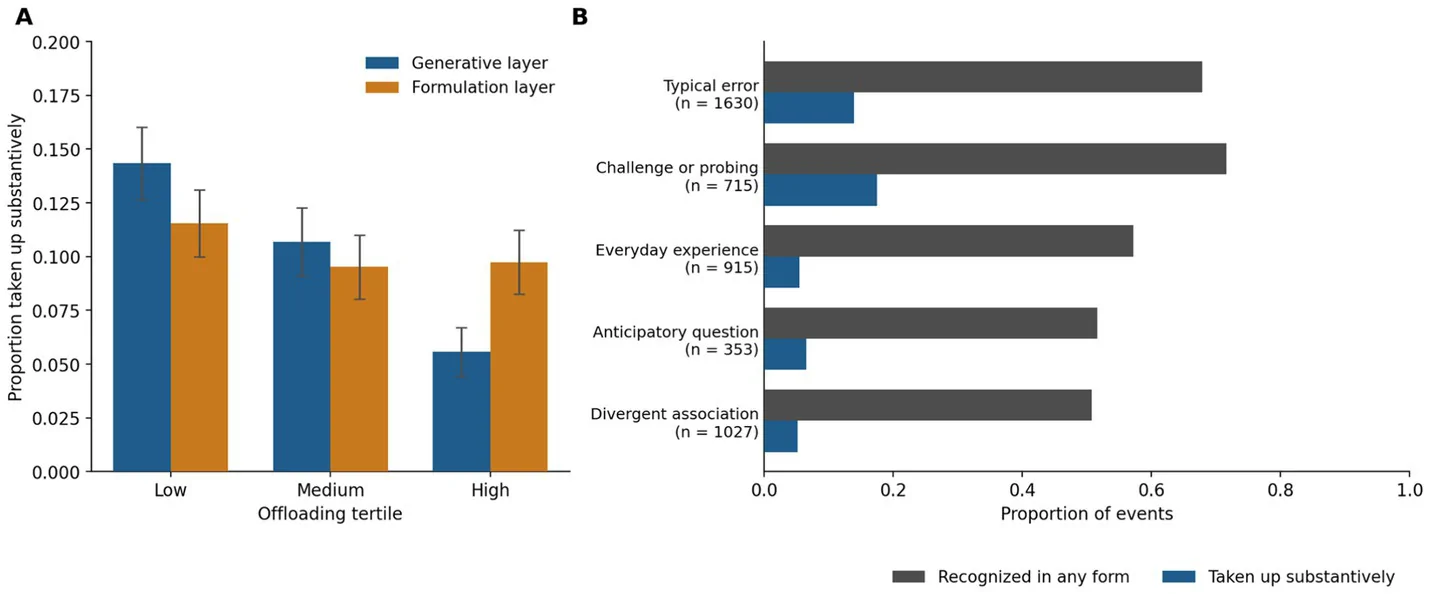
Teacher responses to unplanned student contributions. **(A)** Proportion of events used substantively to advance the lesson, by tertile of generative-layer offloading (blue) and formulation-layer offloading (orange); error bars are 95% confidence intervals. **(B)** Proportion of events recognized in any form and used substantively, by event type in the order used in Table 4, with event counts in parentheses. *n* = 4,640 events in 861 lessons.

### 3.5 Micro-randomized comparison

Random assignment to the generative preservation protocol lowered generative-layer offloading by 0.124 (SE = 0.018, *p* < 0.001; *d* = −0.49) and left formulation-layer offloading unchanged (*b* = −0.022, *p* = 0.272), confirming that the manipulation acted on the intended layer (Table 5). Among distal outcomes, assignment raised substantive uptake by 0.039 (SE = 0.012, *p* < 0.001; *d* = 0.22), with a complier average causal effect of 0.046. Estimates for higher-order questioning (*b* = 0.012, *p* = 0.248), maintenance of cognitive demand (*b* = 0.046, *p* = 0.174), recognition of unplanned contributions (*b* = 0.011, *p* = 0.719), and variation structure were not distinguishable from zero. Minimum detectable effects ranged from 0.18 to 0.29 standard deviations.

**Table 5:** Intention-to-treat estimates from the micro-randomized generative preservation protocol, waves 3 and 4.

Outcome	*b* (SE)	*p*	*d*	*n*	Minimum detectable effect
Generative-layer offloading	−0.124 (0.018)	<0.001	−0.49	427	0.20 SD
Formulation-layer offloading	−0.022 (0.020)	0.272	−0.09	427	0.22 SD
Substantive uptake	0.039 (0.012)	<0.001	0.22	427	0.18 SD
Higher-order questioning	0.012 (0.010)	0.248	0.08	427	0.19 SD
Cognitive demand maintained	0.046 (0.034)	0.174	0.12	427	0.25 SD
Recognition of unplanned contributions	0.011 (0.029)	0.719	0.04	406	0.28 SD
Substantive use of unplanned contributions	0.003 (0.014)	0.831	0.02	406	0.28 SD
Longest variation chain	−0.228 (0.142)	0.107	−0.16	427	0.27 SD
Variation types used	−0.067 (0.074)	0.363	−0.09	427	0.29 SD
Variation chains	−0.170 (0.131)	0.194	−0.13	427	0.29 SD

Assignment covered 154 lessons and implementation covered 130 (84.4%), with no implementation without assignment. Models include teacher random intercepts, topic difficulty, lesson type, and wave. The complier average causal effect for substantive uptake is 0.046. Effect sizes and minimum detectable effects are computed against outcome standard deviations in the wave 3 and 4 subsample in which the trial was run, not the full-sample values in Table 2. The two differ most for higher-order questioning (0.144 against 0.126) and substantive uptake (0.179 against 0.162), both of which have higher means in the later waves. Minimum detectable effects are 2.8 times the estimated standard error divided by that standard deviation, computed from unrounded standard errors.

Those nulls should be read against what the observational models imply the trial could show. The protocol moved generative-layer offloading by 0.124, or 0.71 within-teacher standard deviations, so the intention-to-treat effects implied by the within-teacher slopes in Table 3 run from 0.02 to 0.12 standard deviations across the outcome set. The minimum detectable effects exceed that implied value for every outcome, so the experimental nulls cannot falsify any of the observational estimates. Substantive uptake is the informative case. Its implied effect of 0.12 also falls below the 0.18 threshold, and it was detected anyway, at 0.22.

Multiplying the within-teacher slope for substantive uptake (−0.170) by the shift the protocol produced (0.124) predicts an intention-to-treat effect near 0.021, against an observed 0.039, a difference of about 1.5 standard errors. Three readings are available, and the data do not separate them: the protocol may alter aspects of preparation that our two ratios do not capture, such as the teacher’s own rehearsal of the questioning sequence; the observational slope may be attenuated by measurement error in the ratios; or the difference may be noise. The first is the most consequential for interpretation. The protocol changed the order of preparation, the authorship of the questioning sequence, and the salience of questioning at the same time, so what it identifies is the effect of that protocol rather than the isolated effect of lowering generative-layer offloading. The observational and experimental estimates answer different questions, and the size of the experimental effect is itself a reason to think the protocol carries something the two ratios do not.

Preparation sequence, recorded observationally for every lesson, ordered the same way. Lessons prepared by taking a model draft first and revising it (*n* = 259) had mean generative-layer offloading of 0.591, higher-order questioning of 0.189, and substantive uptake of 0.211. Lessons prepared by drafting first and consulting the model afterward (*n* = 403) had mean generative-layer offloading of 0.210, higher-order questioning of 0.264, and substantive uptake of 0.335. Lessons alternating between the two (*n* = 213) fell between the two at 0.337, 0.232, and 0.294. The remaining 22 lessons were prepared without AI; in those few cases, we can only say that they did not differ appreciably from the draft-first group, and the comparison is too thin to support any conclusion about abstaining from the tool altogether.

### 3.6 Professional attention and subjective preparedness

Within teachers, generative-layer offloading predicted a lower proportion of stimulated-recall statements directed at student thinking (*a* = −0.199, SE = 0.029, *p* < 0.001), while formulation-layer offloading predicted a higher proportion (*a* = 0.140, SE = 0.024, *p* < 0.001). Attention to student thinking in turn predicted substantive use of unplanned contributions (*b* = 0.190, SE = 0.057, *p* = 0.001). The indirect path was −0.038 with a Monte Carlo 95% confidence interval of [−0.065, −0.015], and the remaining direct path was −0.005 (*p* = 0.904) (Figure 3, Table 6).

**Figure 3:**
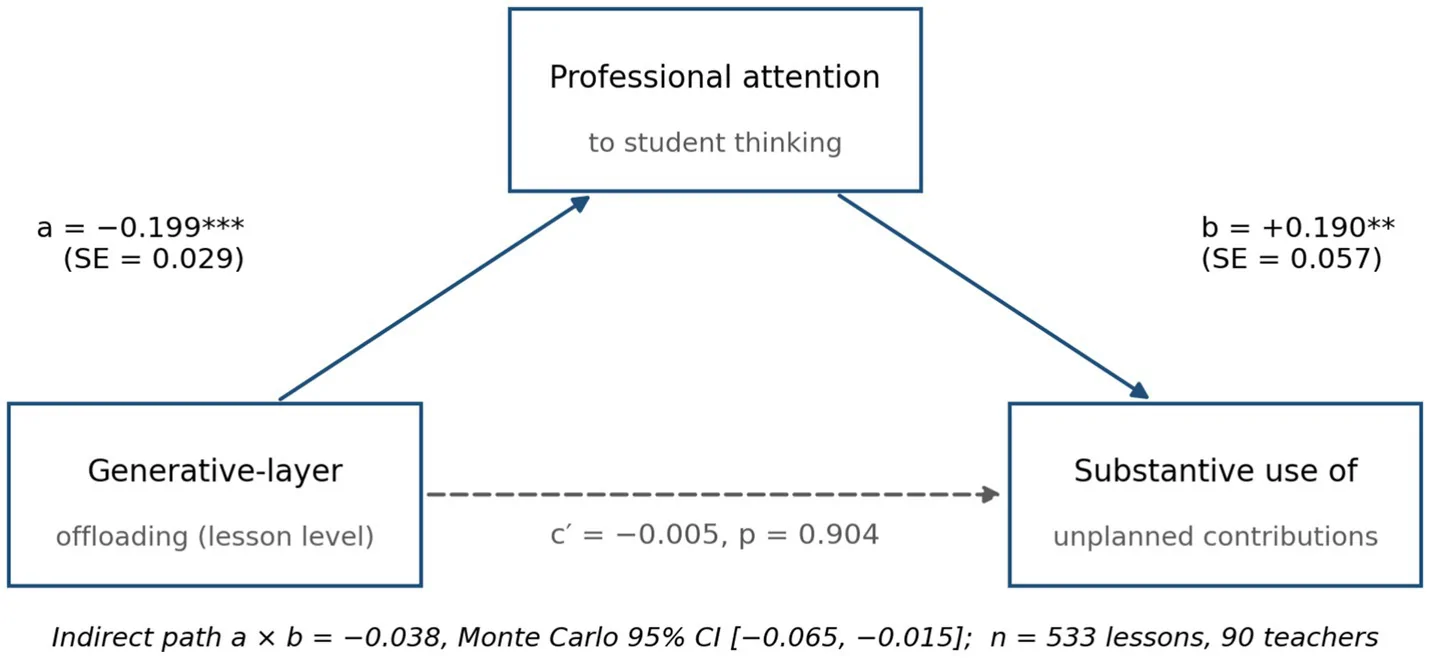
Multilevel mediation model for the within-teacher association between generative-layer offloading and the substantive use of unplanned student contributions. Solid arrows denote significant paths, and the dashed arrow denotes the non-significant direct path. Coefficients are unstandardized. *n* = 533 lessons from 90 teachers. **p < 0.01, ***p < 0.001.

**Table 6:** Multilevel mediation models for the within-teacher association between generative-layer offloading and classroom outcomes, through professional attention to student thinking.

Outcome	*a* (SE)	*b* (SE)	*c*′ (SE)	Indirect [95% CI]	*n*
Substantive use of unplanned contributions	−0.199 (0.029)***	0.190 (0.057)**	−0.005 (0.040)	−0.038 [−0.065, −0.015]	533
Recognition of unplanned contributions	−0.199 (0.029)***	0.341 (0.106)**	−0.220 (0.075)**	−0.068 [−0.118, −0.025]	533
Substantive uptake	−0.216 (0.029)***	0.202 (0.041)***	−0.133 (0.029)***	−0.044 [−0.066, −0.024]	556
Higher-order questioning	−0.216 (0.029)***	0.028 (0.037)	−0.106 (0.026)***	−0.006 [−0.022, 0.010]	556

Path *a* is generative-layer offloading to attention, path b is attention to outcome, and c′ is the remaining direct path. Monte Carlo confidence intervals from 200,000 draws. Total effects in this subsample, obtained as c′ plus the indirect effect, are −0.043 for substantive use of unplanned contributions, −0.288 for recognition, −0.177 for substantive uptake, and −0.112 for higher-order questioning; they differ from the within-teacher coefficients in Table 3 because the mediation models are fitted only on lessons with interview data. Formulation-layer offloading predicted closer attention to student thinking (*a* = 0.140, SE = 0.024, *p* < 0.001). The mediator is measured after the lesson; see Section 4.6. **p < 0.01, ***p < 0.001.

Indirect paths were also present for recognition of unplanned contributions (−0.068, 95% CI [−0.118, −0.025], *n* = 533) and for substantive uptake (−0.044, 95% CI [−0.066, −0.024], *n* = 556), in both cases alongside a direct path that remained significant. For higher-order questioning, the indirect path was small, and its interval included zero (−0.006, 95% CI [−0.022, 0.010]), while the direct path was unchanged (−0.106, *p* < 0.001). The contrast is interpretable. Higher-order questioning is largely fixed when the plan is written, so an association there need not travel through anything that happens in the room. Responsiveness to what students actually say is produced in real time and is gated by what the teacher is attending to.

Subjective preparedness behaved differently again. Within teachers, generative-layer offloading predicted post-preparation confidence (*b* = 1.241, SE = 0.162, *p* < 0.001), an increase of 0.23 standard deviations per within-teacher standard deviation of the ratio, which is the scale on which the classroom coefficients above are reported, and of 1.32 standard deviations across its full range, while formulation-layer offloading did not (*b* = −0.113, *p* = 0.433). Mean confidence rose monotonically across quartiles of generative-layer offloading, from 3.97 to 4.81 (Figure 4A). Confidence carried no positive information about the lesson that followed. Within-teacher correlations with observed indicators were 0.010 for higher-order questioning, −0.008 for substantive use of unplanned contributions, −0.060 for recognition, −0.073 for substantive uptake, and −0.073 for maintenance of cognitive demand, compared with 0.249 for generative-layer offloading itself (Figure 4B). In models controlling only for topic difficulty, lesson type, and wave, confidence weakly predicted lower uptake (*b* = −0.010, *p* = 0.029) and lower maintenance of cognitive demand (*b* = −0.029, *p* = 0.033). Once both offloading layers were entered, confidence predicted no outcome (all *p* > 0.12), so what little it tracked was inherited from the delegation that produced it.

**Figure 4:**
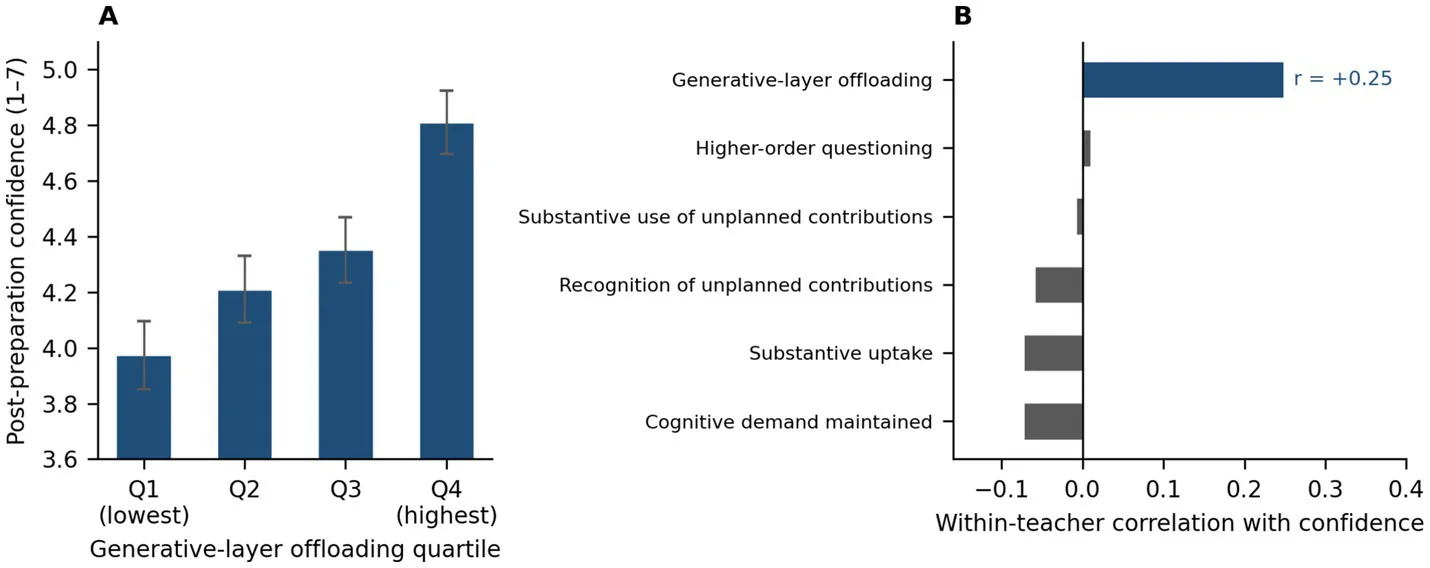
Post-preparation confidence in relation to offloading and to observed lesson quality. **(A)** Mean post-preparation confidence by quartile of generative-layer offloading; error bars are 95% confidence intervals. **(B)** Within-teacher correlations between post-preparation confidence and generative-layer offloading (blue) and five observed classroom indicators (grey). The vertical axis in panel **(A)** is truncated. *n* = 869 lessons for confidence and 833 for the indicators derived from unplanned contributions.

## 4 Discussion

Across 897 lessons taught by 90 teachers over one school year, what teachers delegated to a generative model during preparation predicted the discourse indicators coded from the resulting lesson, and the direction of that prediction depended on which layer was delegated. Handing over the questioning sequence and the ordering of variations went with less higher-order questioning, less substantive uptake, weaker maintenance of cognitive demand, and lower recognition and productive use of unplanned student contributions. Handing over contextual material and linguistic formulation was associated with longer and more varied designed variation, better recognition of unplanned contributions, and no detectable cost on any indicator. Total time with the tool predicted outcomes until layer composition entered the model, after which it predicted none of the lesson-level indicators.

### 4.1 Offloading as a layered construct

The foundational review of cognitive offloading left open whether different forms of offloading are cognitively equivalent, and organized its taxonomy around the destination of the offloaded work (Risko and Gilbert, 2016). For one domain of consequential professional work, our data indicate they are not equivalent, and the measurement is a segment count instead of a self-report of frequency. The count records provenance. A question that came from the model may still have been evaluated, adapted, and rehearsed before it reached the plan, and a question the teacher wrote unaided may have cost very little. Plans whose questioning and variation structure originated in model output were followed by weaker discourse, and offloading is the most economical reading of that. This matters for reading the existing evidence. A widely cited study reporting that AI use diminishes critical thinking through offloading measures both the predictor and the mediator as self-reported quantities (Gerlich, 2025a). Our own data reproduce that association when volume is entered alone and dissolve it when composition is entered. Some part of the reported relation between AI use and thinking may be compositional: heavier users delegate more of everything, including the operations whose delegation carries a cost.

The result gives a field counterpart to the laboratory demonstration that supplying answers and supplying methods produce indistinguishable volumes of help-seeking but different transfer (Spatola, 2024), and it sits alongside evidence that the labeled provenance of guidance makes no difference to how people use it (Pearson et al., 2026). What appears to govern outcomes is the identity of the operation surrendered. On that reading, the difficulty the field has had in detecting stable effects of AI use follows from measuring a quantity where a composition was doing the work.

### 4.2 Why delegating the formulation layer does not carry the same cost

The positive coefficients for formulation-layer offloading are the strongest evidence we have that the partition is real. If teachers who delegate more were simply weaker teachers, or if delegation were a single tendency, selection would push both layers in the same direction. Coefficients of opposite sign on the same lessons are difficult to reconcile with a common cause of that kind. However, they are not impossible: a teacher pressed for time in a given week might sacrifice questioning design to protect materials preparation, and such a trade-off would produce opposite signs with no causal effect. Embedded randomization is what makes that alternative less likely for the generative layer, since assignment moved the ratio without any corresponding change at the formulation layer. A more deflationary reading of the variation result is available, and it can be tested. Formulation-layer offloading is defined by delegating contextual material, while the variation indicators count chains, their lengths, and the types present, which are carried in part by contexts and examples. A model asked for contexts returns contexts; those contexts enter the plan, and they are then counted as variation structure. Were the association produced that way, it should concentrate in contextual variation. It does not. The by-type decomposition in section 3.3 shows the association concentrated in procedural and conditional variation, with contextual variation the weakest of the four and not distinguishable from zero. The types that respond are those whose structure has to be arranged rather than supplied.

Interpretation follows the distinction between processing imposed by presentation and processing that constitutes understanding. Composing wording and assembling contexts are handling costs, and reducing them is a long-standing aim of instructional design (Sweller et al., 2019; Sweller, 2023). Organizing and integrating are the operations that constitute generative processing (Fiorella and Mayer, 2016; Mayer, 2024). When the model absorbed handling costs, the gains appeared in the outcome most demanding of design effort, the length and variety of variation chains, and in the attentional measure. Released capacity is the natural reading of that placement, although what teachers did with the capacity was not itself observed. When the organizing itself was absorbed, the products of organization degraded. Cognitive load theory’s borrowing and reorganizing principle describes how human beings ordinarily acquire information, holding that we obtain most of what we know from others and that doing so imposes less working memory load than random generation (Sweller et al., 2019). This describes a mechanism, and our results do not contradict it; they suggest that when the object of the work is a representation for real-time use with students, rather than an addition to the teacher’s own long-term store, the calculus differs.

The finding bears on the claim that planning belongs with grading among the low-level cognitive tasks machines may assume (García Espinoza et al., 2026). Planning is not one task. Part of it can be delegated with apparent benefit and part cannot, and nothing in the resulting document distinguishes the two.

### 4.3 Attention, and what teachers can detect

The indirect path through attention to student thinking statistically accounted for the association with substantive use of unplanned contributions, part of the associations with recognition and uptake, and none of the association with higher-order questioning. That split is theoretically informative. Questioning structure is largely determined when the plan is written. Responsiveness to a student who says something unanticipated is produced in the moment and is gated by what the teacher is attending to. The component of professional noticing that governs this, deciding how to respond, is also the component that experience alone does not confer (Jacobs et al., 2010), which makes it the capacity least able to absorb being handed away. Attention was recorded after the lesson, which leaves the ordering open and makes noticing measured before preparation the obvious next test (section 4.6). Two accounts that do not run through attention are also consistent with the pattern. Model-generated questions may themselves be cognitively shallower, in which case the association describes the output rather than any change in the teacher. Alternatively, the questions may be sound but harder to enact, because a teacher who did not construct or rehearse a sequence has less of it available in the moment. Neither requires that a capacity was handed away, and the present design does not separate them from the attentional account.

The confidence results suggest why the cost would not be self-correcting. Confidence rose with generative-layer offloading by about a quarter of a standard deviation per within-teacher standard deviation of the ratio. It was uncorrelated with every observed feature of the lesson. This resembles the pattern established for learners, in which manipulating fluency alone moves judgments of learning by about a standard deviation while leaving recall unchanged (Carpenter et al., 2013), and which survives repeated study and test cycles with feedback (Yue et al., 2013). Reviews in that tradition have long asserted that such gains mislead instructors as well as students (Soderstrom and Bjork, 2015; Bjork et al., 2013), but teachers have rarely been examined as subjects of the effect. Preparation with a fluent generative model has the features the illusion requires: a finished-looking product, an experience of rapid progress, and no immediate feedback. The prediction that greater offloading raises metacognitive demand (Tankelevitch et al., 2024), and the expectation that seamless systems lead teachers to mistake the system’s suggestions for their own decisions (Biström and Mollwing, 2026), are both consistent with what we observed. Fluency itself was not measured, and confidence could rise because the plan looks complete, because it lists more contingencies, or because the model is trusted. Isolating these factors would require an experiment.

### 4.4 Relation to divergent findings in the literature

These results suggest a partial resolution of the standing opposition between studies reporting benefits of generative AI in education and studies reporting cognitive costs. Three differences separate the two bodies of work, and the present design removes all three at once. Outcomes measured while the tool is in use differ from outcomes measured after it is withdrawn, reflecting the learning-versus-performance distinction applied across literatures (Soderstrom and Bjork, 2015). Independent variables defined by whether or how much the tool is used differ from independent variables defined by what is surrendered. And outcomes defined as perceptions differ from outcomes defined as observed behavior; defining outcomes as perceptions is a recurring limitation of the acceptance tradition (Xue et al., 2026).

Our data also bear on a well-known null. When six validated dimensions of planning competence failed to predict student-rated cognitive activation, the authors attributed the result to novice status and small samples (König et al., 2021). An alternative that does not require either is available: the pathway from preparation to discourse is detectable when discourse is coded from video, and the earlier null may partly reflect the insensitivity of student perception ratings to conversational behavior. If so, it has a corollary for assessing AI-assisted plans. Scoring systems that award credit for the presence of written elements and stated rationales may overestimate depth of processing under AI conditions, since a generative model can produce a complete and well-reasoned-looking document from a shallow prompt. We did not test this, and offer it as a hypothesis that our design makes testable.

### 4.5 Implications

For practice, the most important finding is about ordering. The randomized protocol that required teachers to draft their own questioning and variation sequence before consulting the model shifted layer composition substantially and improved substantive uptake, and the observational sequence variable ordered the outcomes the same way. Professional development that treats AI use as a quantity to be limited targets a variable that carries no information about the lesson-level indicators once composition is known; development that specifies which operations stay with the teacher targets one that does. Whether abstaining from the tool would do better still is a question our 22 AI-free lessons cannot answer.

For research, systems that return complete, well-formed lesson plans optimize the property most likely to produce misplaced confidence. A system that returned materials and formulations while withholding questioning sequences until the teacher had supplied their own would implement the protocol tested here. For research, instruments that measure AI use as frequency, duration, or reliance capture something that largely vanishes once composition is modeled.

### 4.6 Limitations

The most serious limitation concerns the mediator. Stimulated recall takes place after the lesson, so professional attention was measured later than the outcomes it is used to explain. A teacher who happened to take up student thinking during a lesson has more student thinking to talk about afterward, and retrospective accounts are shaped by the events being recalled. The statistical model treats attention as antecedent; the measurement does not establish that. We attempted a check using the only pre-lesson indicator available, the share of preparation sessions whose dominant request concerned anticipating student thinking and errors. That indicator was unrelated to the stimulated-recall measure (*r* = −0.07) and produced no indirect effect. If a prospective index of attention bears no relation to the retrospective one, the retrospective measure may be registering what happened during the lesson more than a disposition the teacher brought to it. The mediation is reported accordingly, as a mechanism probe.

A related concern applies to one outcome specifically. Higher-order questioning is coded from the lesson, but the questioning sequence largely originates in the plan, and the predictor is the share of that sequence taken from model output. If model-generated questions are themselves cognitively shallower, the association would describe the model’s output rather than any change in the teacher. Our design cannot separate these, because we did not code the cognitive level of questions as written in the plan, and that analysis should be part of any replication. The interpretation is on firmer ground for the outcomes defined over unplanned contributions, since a contribution that departs from the plan cannot have been anticipated in it, and the association there cannot be an artifact of inherited plan content.

The primary estimates are observational. Within-teacher decomposition removes stable teacher characteristics but not time-varying confounders; a teacher under pressure in a particular week might both delegate more and attend less. The embedded randomization addresses this for one outcome, and its identifying assumption is not innocuous: teachers knew which lesson had been designated, so assignment may have affected the lesson through raised salience of questioning as well as through the layer composition it was designed to shift. The complier average causal effect should be read with that in mind.

Our variation coding is an operational segmentation, and its construct status is weaker than that of the questioning and uptake measures. Chain length and diversity are not monotonically good, since variation is discernible only against relative invariance, repetition of same-type items does not by itself constitute variation, and extended sequences may accumulate or merely circle (Watson and Mason, 2006; Pang et al., 2017; Alexander, 2018). The positive association between formulation-layer offloading and chain length indicates more designed variation structure, not demonstrated learning gains. The indicators are selected features of classroom discourse rather than a comprehensive measure of teaching quality, and a lower share of higher-order questions is not by itself evidence of weaker teaching at every stage of a lesson. The absence of a detectable cost for the formulation layer is likewise not evidence of no cost; with 897 lessons, the models would have detected standardized associations of about 0.07 and above.

The candidate moderator does not measure the construct it was intended to measure, so we have no evidence on whether teachers with greater adaptive expertise are protected. Because desirable difficulties can become undesirable when background knowledge is insufficient (Bjork and Bjork, 2011), a genuine moderation test remains motivated and would require dynamic assessment (Schwartz et al., 2005). Finally, the sample comes from one prefecture-level city, one country, one subject, and one phase of schooling, and the study was not preregistered.

## 5 Conclusion

Whether teachers use generative AI to prepare has become an uninformative question, and how much they use it is barely more informative. What predicted the classroom discourse we coded was which layer of preparatory cognition was handed over. Delegating the composition of materials and language went with better-designed variation and no cost we could detect. Delegating the design of questioning and the ordering of variation went with a lesson in which fewer questions demanded analysis, fewer student contributions were taken up, high-demand tasks were more often reduced to procedure, and unanticipated student thinking was more often left unrecognized. In the teacher’s own account of the lesson afterward, that association came with a smaller share of attention directed at student thinking, and with a rise, not a fall, in confidence about handling what students would do. Confidence formed during preparation did not reliably predict the lesson that followed.

## Data Availability

The datasets presented in this article are not readily available because the lesson video and interview audio contain identifiable minors and cannot be shared. The de-identified analysis dataset, the accompanying data dictionary, and the full analysis code are available on request. Requests to access the datasets, including requests for supervised access to the recordings, should be directed to Xin Dang, mafu15091158648@163.com.
